# EcoHIV Infection of Primary Murine Brain Cell Cultures to Model HIV Replication and Neuropathogenesis

**DOI:** 10.3390/v16050693

**Published:** 2024-04-27

**Authors:** Boe-Hyun Kim, Wei Chao, Eran Hadas, Alejandra Borjabad, Mary Jane Potash, David J. Volsky

**Affiliations:** 1 Division of Infectious Diseases, Department of Medicine, Icahn School of Medicine at Mount Sinai, New York, NY 10029, USA; boe-hyun.kim@mssm.edu (B.-H.K.); wei.chao@mssm.edu (W.C.); eran.hadas@mssm.edu (E.H.); alejandra.borjabad@mssm.edu (A.B.); mary.potash@mssm.edu (M.J.P.); 2 Department of Neuroscience, Icahn School of Medicine at Mount Sinai, New York, NY 10029, USA

**Keywords:** HIV infection, neuropathogenesis, primary brain cell cultures

## Abstract

Background. EcoHIV is a chimeric HIV that replicates in mice in CD4+ T cells, macrophages, and microglia (but not in neurons), causing lasting neurocognitive impairment resembling neurocognitive disease in people living with HIV. The present study was designed to develop EcoHIV-susceptible primary mouse brain cultures to investigate the indirect effects of HIV infection on neuronal integrity. Results. We used two EcoHIV clones encoding EGFP and mouse bone marrow-derived macrophages (BMM), mixed mouse brain cells, or enriched mouse glial cells from two wild-type mouse strains to test EcoHIV replication efficiency, the identity of productively infected cells, and neuronal apoptosis and integrity. EcoHIV replicated efficiently in BMM. In mixed brain cell cultures, EcoHIV targeted microglia but did not cause neuronal apoptosis. Instead, the productive infection of the microglia activated them and impaired synaptophysin expression, dendritic density, and axonal structure in the neurons. EcoHIV replication in the microglia and neuronal structural changes during infection were prevented by culture with an antiretroviral. Conclusions. In murine brain cell cultures, EcoHIV replication in the microglia is largely responsible for the aspects of neuronal dysfunction relevant to cognitive disease in infected mice and people living with HIV. These cultures provide a tool for further study of HIV neuropathogenesis and its control.

## 1. Introduction

The principal cellular targets of HIV are CD4+ T lymphocytes, monocyte/macrophages, and microglia. However, HIV can also infect a small fraction of astrocytes in human beings [1,2] and depending upon the strain, SIV replicates well in macaque astrocytes [3]. The infection of astrocytes is limited and aborts prior to the expression of HIV/SIV structural proteins; however, it can modify cellular gene expression [4]. In contrast, there is little evidence that mature neurons are susceptible to HIV infection [5,6], although neural progenitor cells can be infected, particularly in the context of methamphetamine [7,8]. A critical distinction between HIV immunodeficiency and brain disease is the cellular origin of injury. HIV directly infects and kills T lymphocytes, causing profound immune dysfunction, but it appears to impair neuronal function indirectly, through products of infected cells and through impaired neuronal support from infected cells [9,10].

Here, we propose to quantify HIV infection and aspects of neuronal injury and morphological changes in microglia in well-defined target cell types, including primary mixed murine brain cell cultures, using a well characterized chimeric HIV, EcoHIV [11]. EcoHIV carries ecotropic murine leukemia virus envelope gp80 in place of gp120, switching HIV host tropism from human beings to rodents [11]. In rodents, EcoHIV infects CD4+ T cells, macrophages, and microglia, causing neurocognitive impairment, synaptodendritic injury in the hippocampus and cortex, and profound dysregulation of gene expression in the brain [11,12,13,14,15,16]. Within the lymphoid compartment, only CD4+ T cells are susceptible; B lymphocytes and CD8+ T lymphocytes, are not infected [11]. In peritoneal cells from infected mice, F4/80+ macrophages carry the bulk of virus [13]. In the brain, macrophage/microglia have been shown to be infected by EcoHIV in mice and rats [14,16]; however, the possibility that other brain cells may also be infected, thereby impairing neuronal function, has not been directly addressed.

We approached this possibility by initiating EcoHIV infection in a variety of cell types in culture, identifying infected cells by microscopy using dual fluorescent tags for cell type and productive infection. We also probed the dysfunction in neuronal protein expression and changes in microglia morphology during the infection in primary cultures to identify the routes to brain disease. Our results confirm those of previous studies in EcoHIV-infected mice, demonstrating the productive infection of macrophages and microglia and showing that within mixed brain cell or glia cultures, only microglia are infected, and that this infection activates microglia and causes a significant reduction in the number of dendrites per neuron. We also find that the EcoHIV infection of microglia in these cultures and the consequent neuronal injury can be prevented by antiretroviral treatment. Studies presented here regarding EcoHIV infection and pathogenesis in primary brain cells complement previous studies in infected animals and conserve investigator effort and research reagents, as well as minimize animal euthanasia. This system is a convenient culture model for further investigation of HIV replication and control within the brain.

## 2. Materials and Methods

Mice, cells, and tissue culture. All experiments using mice as cell donors were conducted with the approval of the Icahn School of Medicine at Mount Sinai Institutional Animal Care and Use Committee. Male and female 129x1/SVJ (strain #000691) and C57BL/6J (strain #000664) mice were purchased from the Jackson Laboratory, Bar Harbor, ME, USA. To obtain embryonic and neonatal mice, we co-caged male and female mice, selected pregnant females by detection of a vaginal plug within 16 h, removed the pregnant mice, and began timing fetal age. For enriched glial cell culture, the cortex was collected from 1-day neonates, and meninges were removed under a dissecting microscope. Cells were mechanically isolated using a glass Pasteur pipette and cultured in DMEM/F12 with 10% FBS and 1% penicillin-streptomycin (ThermoFisher Scientific, Waltham, MA, USA) in 100 mm dishes and 1 mm^2^ chamber slides until cells grew to confluence, for about 3 weeks. Glial cells were collected from a 1 mm^2^ chamber slide to count the total number of cells; the average number of glial cells in a 1 mm^2^ chamber slide was 4.84 × 10^6^ cells. The ratio of microglia cells and astrocytes was measured by immunofluorescent staining using cell markers CD11b and GFAP, respectively [17,18].

For neuronal cell cultures, vessels were aseptically coated with 1.0 mL/25 cm^2^ poly-D-lysine (ThermoFisher Scientific, cat# A3890401) and rocked gently to ensure even coating. After 5 min, the solution was removed by aspiration and allowed to air dry for at least 12 h before culture. The meninges were removed from 14-day-old embryos and the cortex and hippocampus were collected under the dissecting microscope. Cells were mechanically isolated using a glass Pasteur pipette and cultured in Neurobasal medium with 2% B-27 and 2.5S NGF (50 ng/mL) for 3 h, and then the medium was changed. A total of 30% of the medium was changed every 3 days [17,18]. 

For mixed brain cell culture, when the glial cell culture reached confluence, about 20 days after seeding, the glial cell medium was replaced with the Neurobasal medium. After 24 h, isolated neurons were collected from the culture wells, and 3.1 × 10^5^ cells (6%) of the neurons were seeded on confluent glial cells. Under these conditions, neuronal adhesion and neurite growth on glial cells occur at around 6–7 days [18]. For mouse bone marrow-derived macrophage (BMM) culture, 8–10-week-old mice were sacrificed by carbon dioxide asphyxiation; marrow was harvested from separate hind legs; erythrocytes were lysed using ACK lysing buffer (Lonza, Walkersville, MD, USA) for 5 min at room temperature; and nucleated cells were cultured in RPMI1640 with 10% horse serum, 5% fetal bovine serum, 100 units/mL penicillin/streptomycin (ThermoFisher Scientific), and 20% L929 conditioned medium [19]. The MLV-susceptible XC rat tumor cells (ATCC^®^CCL-165^TM^, Manassas, VA, USA) were cultured in DMEM with 10% FBS and 100 units/mL penicillin/streptomycin.

EcoHIV construction, preparation, and infection. Ecotropic murine leukemia virus (MLV) [20] and GFP-expressing MLV (MLV-GFP) were kindly provided by Drs. Y. Sabo and S. Goff, Columbia University. The MLV stocks were confirmed for infectivity in rat XC cells [21] and normalized for infection to EcoHIV stocks by QPCR amplifying shared *env* RNA [13,22]. Infectious EcoHIV/NL4-3-EGFP and EcoHIV/NDK-EGFP were constructed by an insertion of an EGFP cassette into EcoHIV/NL4-3 or EcoHIV/NDK, as previously described [23]. Please note that the EGFP cassette contains an initiation codon and an IRES; it is inserted upstream of the intact *nef* gene [23]. Chimeric viruses were washed and resuspended in saline for infection. For viral infection, cells were cultured in complete culture medium without antibiotics 24 h before infection and exposed to the virus in culture medium without serum and antibiotics for 3 h; then, the complete culture medium was replaced, and the cultures were incubated in 5% CO_2_ at 37 °C for 3–14 days. Unless otherwise specified, the cultures were infected at a dose of 1.0 pg p24 per cell for a period of 7 days.

Quantitative real-time RT-PCR (QPCR). For QPCR, RNA was isolated from the cells by using a modified TRIzol (ThermoFisher Scientific) protocol [11]. The expression of selected genes in the samples was examined by using Taqman chemistry, with MGB probes and primers selected from the Applied Biosystems assay and Roche on-demand programs (Supplementary Table S1). The relative efficiency of all assays was compared to GAPDH and was within the parameters established for ΔΔCt (threshold cycle) analysis. The test transcript values were normalized to levels of GAPDH and presented as fold change or as absolute expression levels. Each experiment was repeated at least three times.

Immunological methods. The HIV p24 content in cell supernatants was measured by ELISA, according to the manufacturer’s instructions (HIV Ag kit, Coulter, Brea, CA, USA). For staining, cells were fixed with 4% paraformaldehyde in PBS, permeabilized with 0.2% Triton X-100 (Sigma-Aldrich, Burlington, MA, USA) at 4 °C for 10 min, treated with 5% normal donkey serum (Jackson ImmunoResearch, West Grove, PA, USA) in PBS at R.T. for 1 h, and washed with PBS [17,18]. The cells were incubated with anti-GFP (1:500, ThermoFisher Scientific), anti-CD11b (1:100, BD Pharmingen, Franklin Lakes, NJ, USA), anti-GFAP (1:100, Cell Signaling, Danvers, MA, USA), anti-MAP2 (1:100, Millipore, Burlington, MA, USA), anti-SYP (1:10, Abcam, Cambridge, UK), β-actin (1:1000, Sigma-Aldrich), and anti-p24 (1:50, H12 clone, NIAID, Rockville, MA, USA). 

For the secondary antibodies, anti-chicken Alexa 488 or anti-rabbit Alexa 488 (1:200; ThermoFisher Scientific), anti-rabbit Alexa 555, anti-mouse Alexa 555 (1:100; ThermoFisher Scientific), or anti-rat Alex 405 (1:100) were added to slides in PBS for 1 h and washed with PBS three times. DAPI (ThermoFisher Scientific, cat# P36941) staining was used as a nuclear marker, and cells were counted under an Axiovert 200 fluorescence microscope (Zeiss, Oberkochen, Germany) for statistical analysis. Briefly, 1 × 10^6^ cells which were positive for DAPI and the designated cell lineage marker were observed and the EGFP-positive cells were counted, then calculated and presented as the percentage of EGFP positive cells in a given cell population using Velocity 6.0 (PerkinElmer, Waltham, MA, USA). Axonal thickness, dendrite numbers/cell, and the fluorescence intensity of SYP were measured using the ImageJ program (NIH free image analysis software). 

For axon, dendrites, and SYP image analysis, 50 cells were counted from each group for statistical analysis. For p24 staining, a catalyzed signal amplification (CSA) system (Dako, Glostrup, Denmark) was used, employing H12 monoclonal antibody, and then the cells were inspected using upright microscopy (Nikon, Minato City, Japan). For the measurement of microglial length, a total of 10 different fields from three independent experiments were selected, measuring the longest axis of each cell. Then, the average of the longest and the shortest length was calculated from each group and presented in the graph using Velocity 6.0 software. The experiments were repeated at least three times.

TUNEL assay for cellular apoptosis. The cells were fixed on a cell culture glass chamber slide (Nalgen Nunc, Rochester, NY, USA) with 4% paraformaldehyde in PBS, then permeabilized with 0.2% Triton X-100 (Sigma-Aldrich) at 4 °C for 10 min, treated with 5% normal donkey serum (Jackson, USA) in PBS at room temperature for 1 h, and washed with PBS [17]; cell death was determined using the TACS^®^XL Blue Label Kit (Trevigen, cat# 4828-30-BK, Gaithersburg, MD, USA) and following manufacturer’s protocol. The positive control was generated by treating the sample with TACS-Nuclease^TM^ to generate DNA breaks in the cells. The samples were analyzed under an upright microscope (Nikon), and the images were acquired using a Leica DFC425 camera. Image analysis was conducted by LAS V4.0 (Leica, Wetzlar, Germany) by counting positive-staining cells per 1 mm^2^ of 10 different fields from each of three separate batches of slides, for a total of 30 fields for each group.

Antiretroviral drug inhibition analysis. For mixed brain co-culture, 6–7 days after seeding, neuronal cells were added to the cultures of enriched glial cells, as described above. Cultures were treated with 1 µm of Abacavir (ABV) (Sigma-Aldrich, cat# SML0089) in complete medium without antibiotics. A total of 24 h after ABV exposure, the cells were infected with EcoHIV for 3 h, washed, and then cultured in complete medium with ABV. Every 3 days, 30% of the medium containing ABV was replaced. A total of 7 days after infection, the cells were fixed with 4% PFA for immunofluorescent staining.

Statistical analysis. Quantitative analyses were performed by *t*-test (Sigma Plot version 15). For statistical evaluation, we used 8 four-chamber slides for XC cell culture, 29 four-chamber slides each for BMM and rich glial cell culture of 129x1/SVJ and C57BL/6J, and 30 four-chamber slides for each mixed brain culture of 129x1/SVJ and C57BL/6J. For drug inhibition, a total of four two- and four-chamber slides each were used for the C57BL/6J mixed brain culture. A total of 188 four-chamber slides and 4 two-chamber slides were used for the statistical evaluation of fluorescent microscopy. The intensity of the fluorescence was measured using Image J software. All data are reported as means ± SEM. Differences were considered significant at a value of ** *p* < 0.05. Comparisons that reached the significance level of *p* < 0.01 were designated with * or #; *p* < 10^−6^ was designated with †; *p* < 10^−8^ was designated with %; and *p* < 10^−20^ was designated with §.

## 3. Results

**Figure 1:**
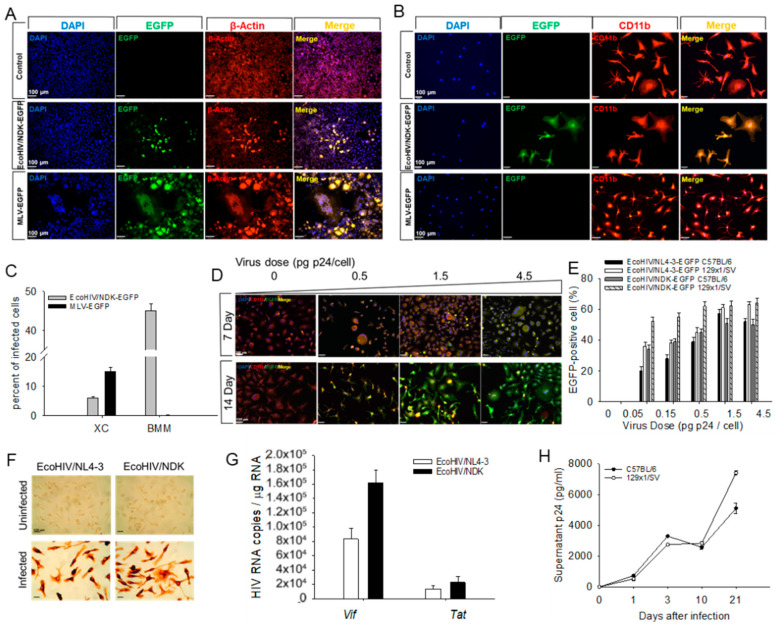
EcoHIV clone and dose, mouse strain, and time-dependent infection of BMM in culture. (**A**–**C**) (**A**) XC cells or (**B**) C57BL/6J BMM were infected with EcoHIV or MLV at a dose of 0.1 pg p24 per cell or the equivalent and (**C**) cells were stained for EGFP (green) and cellular marker β-actin (red), or macrophage marker CD11b (red) and DNA stain DAPI (blue), and the number of infected cells was counted. (**D**) Dose–response of EcoHIV/NDK-EGFP in C57BL/6J BMM. Cells were infected at the indicated doses, and virus expression was measured as described above. (**E**) C57BL/6J or 129x1/SVJ BMM were infected with EcoHIV/NDK-EGFP or EcoHIV/NL4-3-EGFP at doses indicated, and EGFP positive cells were counted 7 days after infection. (**F**,**G**) C57BL/6J BMM were infected with EcoHIV/NDK-EGFP or EcoHIV/NL4-3, and 7 days after infection, were (**F**) stained for p24 or (**G**) subjected to QPCR amplifying *tat* or *vif* RNA. (**H**) BMM of C57BL/6J or 129x1/SVJ mice were infected with EcoHIV/NDK, as indicated, and culture supernatant p24 was measured by ELISA.

Comparison of EcoHIV and MLV infection of mouse and rat cells in culture. In mice, EcoHIV largely reproduces the tropism of HIV, productively infecting CD4+ lymphocytes, macrophages, and microglia [11,13,14]. This tropism differs from that of its envelope donor, ecotropic MLV, which mainly transforms CD4+ lymphocytes in mice [24,25]. In Figure 1, to probe viral target cells in culture, we employed rat sarcoma XC cells, often used to titer MLV infectivity [21], and primary murine BMM, which are reliably susceptible to EcoHIV [26]. Throughout the present study, infection was performed using EcoHIV expressing EGFP during productive infection, here employing clade B EcoHIV/NL4-3-EGFP and clade D EcoHIV/NDK-EGFP, also called EcoNL4-3 and EcoNDK, respectively. Both BMM and XC cells were susceptible to both EcoHIV clones (Figure 1A–C); however, they differed in the extent of their susceptibility, with 6% XC cells and 45% BMM positive for EcoHIV/NDK-EGFP (Figure 1C). In contrast, MLV failed to replicate in BMM (Figure 1A,B), consistent with its inability to productively infect resting cells [27]. The infection by EcoNDK was dose-dependent, and the progeny virus was infectious, as indicated by the increase in EGFP positive cells over time (Figure 1D). The BMM from C57BL/6J and 129x1/SVJ mice were susceptible to both EcoNL4-3 and EcoNDK, with more than 50% of cells of each strain infected by each virus at a dose of 1.5 pg p24 per cell (Figure 1E). Consistent with Figure 1A,B, BMM infected by either EcoNL4-3 or EcoNDK produced p24 (Figure 1F), which increased over time after infection (Figure 1H). The measurement of singly spliced (*Vif)* or multiply spliced (*Tat*) RNA indicates that both transcripts are expressed by both viruses, with EcoNDK being more productive.

**Figure 2:**
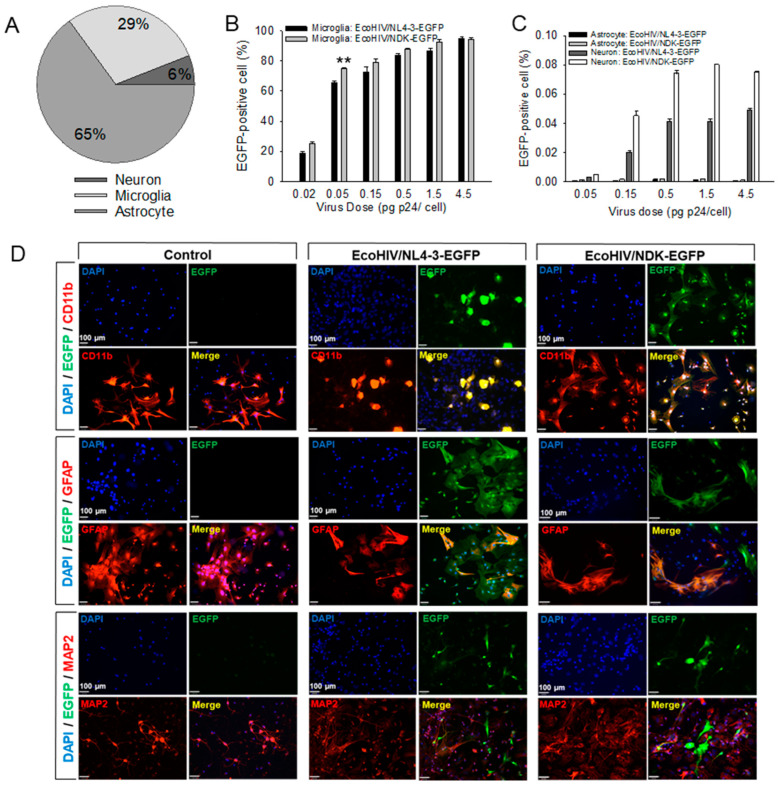
EcoHIV/NDK-EGFP and EcoHIV/NL4-3-EGFP infect predominantly microglial cells in mouse mixed brain cell cultures. (**A**) Cells were stained for nuclear marker DAPI (blue), microglia marker CD11b (red), and viral marker EGFP. (**B**,**C**) Cell composition of mixed brain cell cultures. Frequency of infection and dose–response to EcoHIV/NDK-EGFP or EcoHIV/NL4-3 in different cell types: (**B**) CD11b stained microglia ** *p* < 0.05, EcoHIV/NL4-3-EGFP vs. EcoHIV/NDK-EGFP, (**C**) MAP2 stained neurons or GFAP stained astrocytes. Please note differences in the Y-axis scales in (**B**,**C**). (**D**) Visualization of EcoHIV/NDK-EGFP or EcoHIV/NL4-3-EGFP CD11b-stained microglia, GFAP-stained astrocytes, and MAP2-stained neurons. Scale bar = 100 μm.

Comparison of cell types infected by EcoHIV in mixed brain cell culture. Mixed brain cell cultures were established by adding purified cultured neurons to established enriched glial cells cultures (See Section 2 for details). The cultures were either left uninfected or were infected with EcoHIV/NL4-3-EGFP or EcoHIV/NDK-EGFP at the indicated doses of p24, cultured for 7 days, and stained for EGFP, to label infected cells, and either CD11b, GFAP, or MAP2, to label microglia, astrocytes, or neurons, respectively. The cultures contained mostly astrocytes, 65%, with 29% microglia and 6% neurons (Figure 2A). Over 90% of microglia were infected at the highest virus dose, 4.5 pg p24 per cell, with EcoNDK slightly more infectious than EcoNL4-3 at lower virus doses (Figure 2B). However, the viruses infected fewer than 0.08% of neurons or astrocytes, with little evidence of dose response (Figure 2C, please note Y axis scale). Figure 2D shows representative fields stained by CD11b to visualize microglia infected at 1 pg p24 per cell. Also, we provide images of EGFP-positive astrocytes or neurons infected at this virus dose. These are not representative fields but fields in which we detected any of the rare (<0.08%) astrocytes or neurons stained for EcoHIV-EGFP. These findings of minimal astrocyte or neuron infection concur with the results of studies of brain tissue from EcoHIV-infected mice or rats [14,16]. 

**Figure 3:**
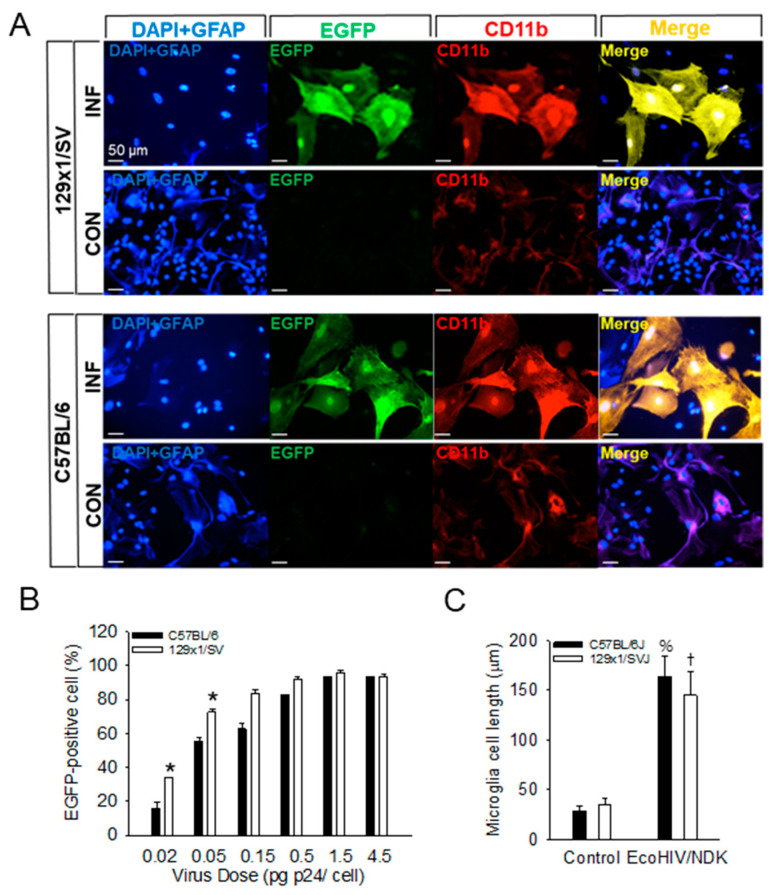
Microglia are the major target of EcoHIV in both 129x1/SVJ and C57BL/6J glial cell cultures. (**A**) Enriched brain glial cell cultures from C57BL/6J and 129x1/SVJ mice were exposed to EcoHIV/NDK-EGFP, and infection in microglia or astrocytes was visualized by immunofluorescence staining for HIV-EGFP (green), macrophage/microglia marker CD11b (red), astrocyte marker GFAP (blue), and nuclear marker DAPI (blue). Scale bar = 50 μm. (**B**) Dose–response to EcoHIV/NDK-EGFP infection of microglia *: *p* < 0.01 C57BL/6J vs. 129x1/SVJ. (**C**) Length of microglia in infected cultures shown in (**A**) †: *p* < 10^−6^ 129x1/SVJ uninfected vs. EcoHIV/NDK-infected, %: *p* < 10^−8^, C57BL/6J uninfected vs. EcoHIV/NDK-infected.

Comparison of susceptibility of mouse strains to EcoHIV in enriched glial cultures. Enriched glial cell cultures were prepared from brains of one-day-old 129x1/SVJ or C57BL/6J mice, and the experiment described in Figure 2 was repeated, infecting cultures with EcoHIV/NDK-EGFP (Figure 3). As in the experiment shown in Figure 2, microglia were productively infected (Figure 3A), but fewer than 0.02% of astrocytes were infected at a dose of 1 pg p24 per cell (please see Figure 2C). Microglia from both mouse strains were infected in dose–response manner to virus, with 129x1/SVJ cells slightly more susceptible than C57BL/6J cells (Figure 3B). In addition, microglia from both strains were enlarged significantly. The microglial lengths from uninfected C57BL/6J cultures averaged 28.8 ± 5.3 µm, and those from 129x1/SVJ averaged 35.4 ± 6.3 µm. However, in infected C57BL/6J cultures, microglial lengths averaged 163.3 ± 21 µm), and those in infected 129x1/SVJ cultures averaged 144.6 ± 23.4 µm (please see Table S2). These findings indicate that EcoHIV susceptibility is not restricted to one mouse strain and show that infection causes extensive microglial enlargement, as reported by the activation and inflammatory responses in the brain [28,29,30].

**Figure 4:**
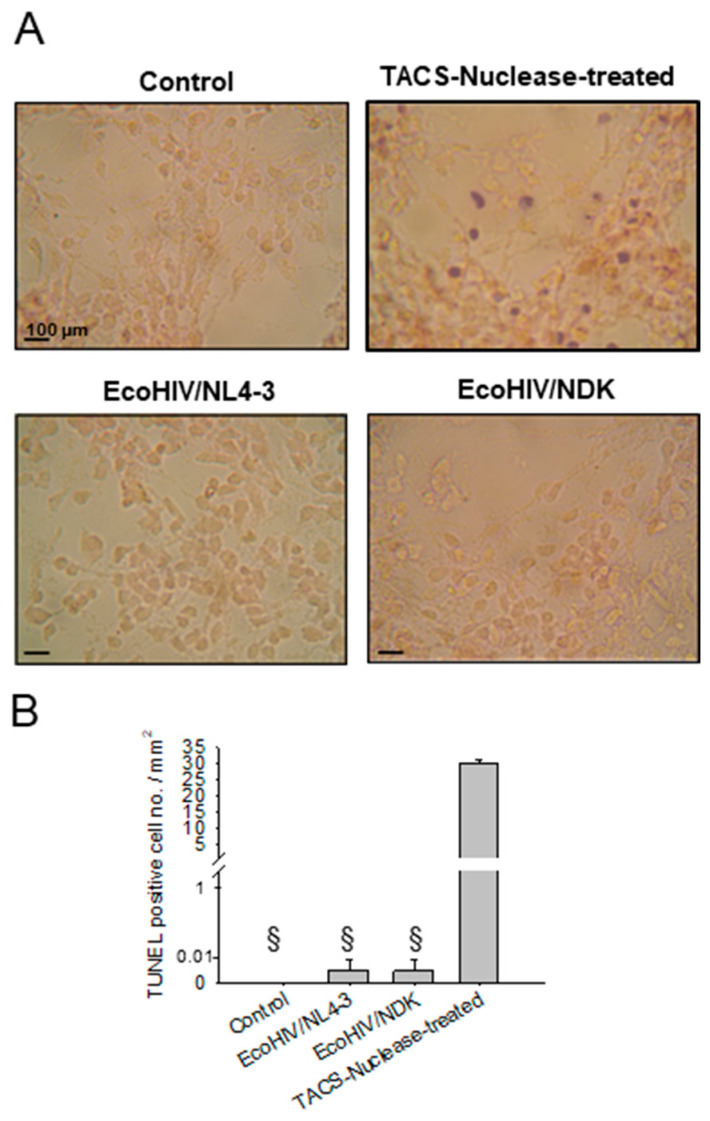
Apoptosis in EcoHIV-infected brain cell cultures. Mixed brain cell cultures from C57BL/6J mice were infected with the indicated virus for 7 days at 1 pg p24 per cell. (**A**) Apoptotic cell death was detected by TUNEL assay; TACS-nuclease-treated cells serve as positive controls. Scale bar = 100 μm. (**B**) Quantitation of apoptotic cell death: §: *p* = 10^−20^ TACS nuclease vs. control, EcoHIV/NDK infected, or EcoHIV/NL4-3 infected.

Neuropathogenic events during EcoHIV infection of mixed brain cell cultures. In infected mice, EcoHIV reproduces several HIV neuropathological events observed in brain tissues from people living with HIV (PLWH) who died with HIV-associated neurocognitive impairment (NCI), including synaptodendritic injury, downregulation of genes involved in neuronal signal transmission, increased brain glutamate levels, and dysregulation of brain lipid metabolism [13,14,15,16,31,32]. Apoptosis and neuronal loss have been observed in brain tissue obtained at autopsy from HIV patients who died with HIV encephalitis [33], but it is not a major finding from the brains of PLWH manifesting NCI [34] or in EcoHIV-infected mice with NCI [14]. To evaluate EcoHIV-associated apoptosis in culture, we performed a TUNEL assay using mixed brain cell cultures infected with either EcoHIV/NDK or EcoHIV/NL4-3; negative controls were uninfected cultures, and positive controls were treated with TACS nuclease (Figure 4). TACS-nuclease-treated cultures showed 30 TUNEL-positive cells/mm^2^; however, neither infection by EcoHIV/NDK nor EcoHIV/NL4-3 induced apoptosis in brain cell cultures, and infected cultures resembled uninfected cultures (Figure 4B). The absence of apoptosis observed here concurs with the measurements of apoptosis in the brain, performed in our previous studies of neurocognitive impairment in mice following EcoHIV infection by intracranial injection [14]. 

**Figure 5:**
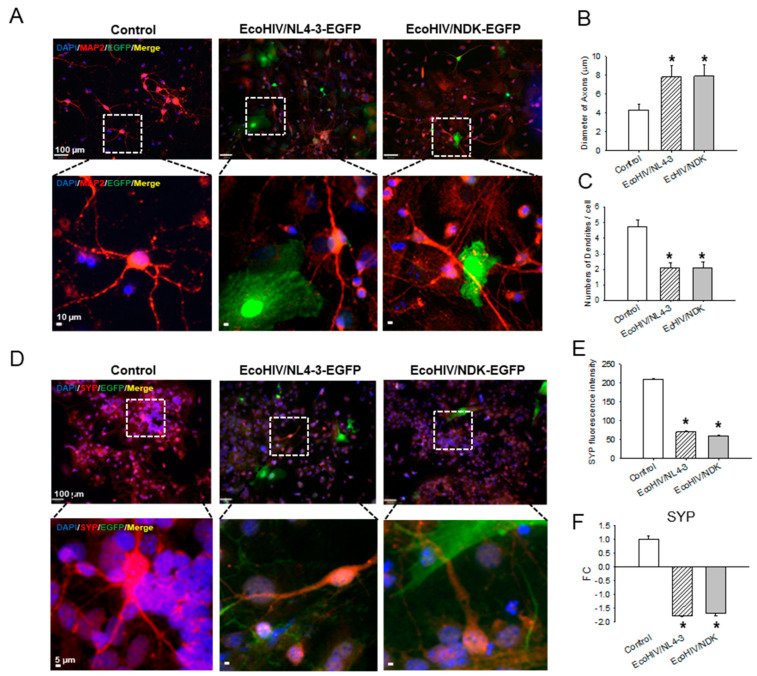
Changes in neuronal morphology in EcoHIV-infected mixed brain cultures. Mixed brain cell cultures from C57BL/6J mice were infected with the indicated virus for 7 days at 1 pg p24 per cell. (**A**–**C**) Cells were stained for neuronal marker MAP2 (red), viral marker EGFP (green), and DNA marker DAPI (blue). Scale bars = 100 μm (upper) and 10 μm (lower) panels. (**A**) MAP2-stained axons and dendrites were visualized: (**B**) axonal thickness and (**C**) dendrite number were measured using ImageJ software. (**D**–**F**) Cells were stained for neuronal SYP (red), viral marker EGFP (green), and DNA marker DAPI (blue). Scale bars = 100 μm and 5 μm. (**E**) Quantitation of SYP protein expression levels using ImageJ software. (**F**) SYP RNA expression level by QPCR. *: *p* < 0.01 uninfected vs. HIV infected.

Since neurons appear to survive EcoHIV exposure in the brain or in culture, but infected mice show cognitive impairment, in experiments shown in Figure 5, we investigated whether EcoHIV infection of mixed brain cell cultures reproduces the synaptodendritic injury observed in vivo [14,15,35,36]. We measured the axonal thickness in MAP2-stained neurons and found that EcoNDK and EcoNL4-3 infection of mixed cell cultures showed an increased axonal thickness from roughly 4.3 μm in uninfected cultures to about 7.8 μm in infected cultures (Figure 5A,B). Moreover, the neurons in EcoHIV-infected cultures also displayed reduced numbers of dendrites compared to uninfected cultures, as shown in the representative images in Figure 5A,D and quantified in Figure 5C. In uninfected cultures, neurons carried, on average, five dendrites each, while EcoHIV infection reduced that to two dendrites per neuron (Figure 5C). Similarly, neuronal expression of the presynaptic zone protein synaptophysin (SYP) was reduced in EcoHIV-infected cultures compared to uninfected cultures (Figure 5D,E). The intensity of SYP staining in neurons in the control cultures was roughly 210, while the intensity of staining in neurons in EcoHIV/NL4-3 infected cultures was about 70 and in neurons from EcoHIV/NDK-infected cultures, it was about 61 (Figure 5E). The defect in SYP expression appears to be imposed, at least in part, at transcription; SYP RNA from EcoHIV-infected cultures was reduced 1.7–1.8-fold compared to uninfected cultures. Taken together, these defects are consistent with our evaluation of synaptodendritic injury in infected mice [14,15], and they may reflect the impaired learning observed in EcoHIV-infected mice.

**Figure 6:**
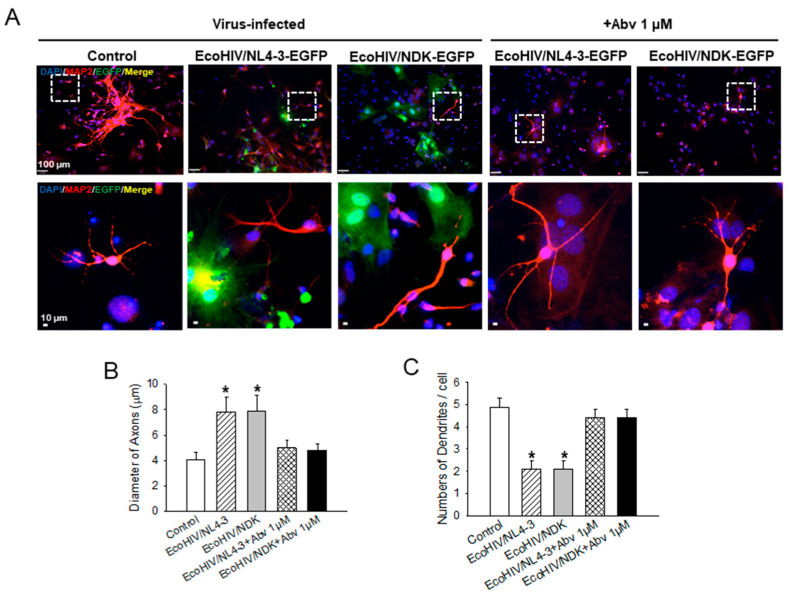
Inhibitor of HIV reverse transcription, ABV, prevents EcoHIV/NDK-EGFP and EcoHIV/NL4-3-EGFP infection and neuronal dysfunction in mixed brain cell cultures. Cultures were treated and infected as described in Section 2. (**A**) MAP2-stained axons and dendrites were visualized. White boxes in the upper panels were enlarged in the lower panels. (**B**) axonal thickness and (**C**) dendrite number were measured using ImageJ software—DNA marker DAPI (blue), neuronal marker MAP2 (red), and viral marker EGFP (green). Scale bars = 100 μm (upper panels) and 10 μm (lower panels). *: *p* < 0.01 uninfected vs. HIV infected or Abv-treated vs. HIV infected.

Isolated HIV gp120 envelope protein can impair neuronal function and kill neurons in tissue culture [37]. EcoHIV does not express gp120, but it clearly impairs neuronal functions in infected mice [13,14,15], as well as neuronal integrity in the mouse mixed brain cell cultures evaluated here. We investigated whether the neuropathogenic effects of EcoHIV observed in the present work (Figure 3, Figure 4 and Figure 5) require virus replication, presumably in microglia, or whether binding of the EcoHIV envelope MLV gp80 is sufficient. Mouse mixed brain cell cultures were established, as shown in Figure 2 and Figure 5, and left untreated or treated with 1 μM of the reverse transcription inhibitor, abacavir (ABV) [38], 24 h prior to EcoNL4-3 or EcoNDK infection. ABV was maintained throughout infection. As shown in Figure 6, infection of mixed brain cultures with either EcoHIV/NDK-EGFP or EcoHIV/NL4-3-EGFP resulted in a thickening of the axons and fewer dendrites displayed by neurons compared to those in uninfected cultures, confirming the alteration of neuronal morphology in EcoHIV-infected cultures (see also Figure 5). Please note that neurons were not directly infected; MAP2 positive neurons were not stained by EcoHIV-EGFP (Figure 6A). However, ABV terminated EcoHIV reverse transcription, presumably in microglia, and allowed for the maintenance of healthy neuronal morphology, including axonal thickness and the density of the dendrites. Thus, in murine brain cell cultures, the productive EcoHIV infection of microglia is required to indirectly impair neuronal integrity, as observed in infected mice [13,14].

## 4. Discussion

Studies using archived brain tissues from HIV patients who died with HIV encephalitis demonstrated unequivocally that HIV productively infects perivascular macrophages and microglial cells in the brain parenchyma, in many cases producing the characteristic “giant cells” [35]. HIV also establishes non-productive infection in a small subset of astrocytes that can arrest prior to or following reverse transcription [2]. Neurons are not infected, but the consensus view is that infected brain macrophages/microglia affect neuronal function indirectly through the production of viral and cellular neuropathogenic proteins, which affect neuronal integrity and eventually, survival [34]. Brain macrophages/microglia are also the main cellular substrates of chronic HIV infection in the central nervous system of PLWH on sustained antiviral therapy, but they carry the virus at a much lower frequency than the levels noted in patients with HIV encephalitis [39,40,41]. Lower levels of brain HIV in PLWH are likely responsible for the milder, chronic, and largely non-degenerative course of NCI in these patients compared to those with HIV dementia [5,10,34,42]. Clarifying the routes to HIV brain disease in the currently predominant non-dementia forms of HIV-associated NCI is a critical experimental goal.

**Figure 7:**
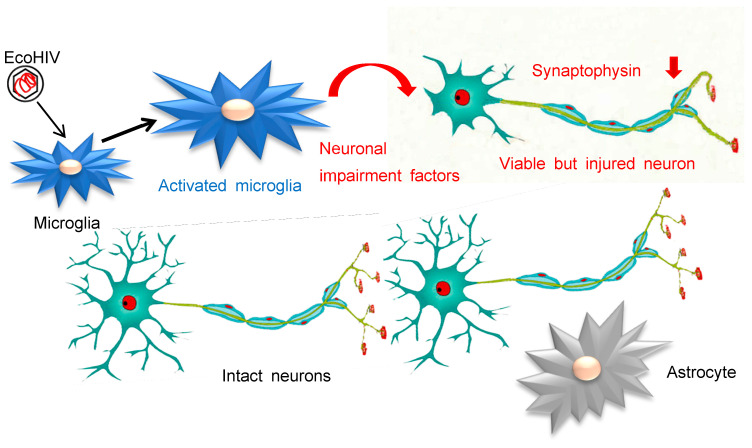
Scheme of EcoHIV infection and pathogenesis in mouse brains.

We have previously developed a tool to approach this goal by constructing chimeric HIV, EcoHIV, that infects rodents and causes HIV-NCI-like disease in mice [11,13,14,15,43]. Here, we demonstrate that EcoHIV infects microglia in a mixed mouse primary brain culture system, allowing for the modeling of aspects of HIV brain disease, especially the indirect injury to neurons caused by infected microglia illustrated schematically in Figure 7. The ability to employ primary mouse brain cell cultures in this pursuit reduces the need to infect and euthanize individual animals for some mechanistic studies or to utilize human stem cells that must be treated with growth/differentiation factors to develop the desired cell types [44]. Also, EcoHIV/NDK and EcoHIV/NL4-3 that encode EGFP or luciferase [13] provide versatile viral markers to identify productively infected cells in a variety of formats. We find that like HIV, EcoHIV highly efficiently infects primary macrophages, but it is less infectious in rat tumor cells. Using mixed brain cell and enriched glial cell cultures established from fetal or newborn mouse brains, respectively, we demonstrate that mainly microglia and not astrocytes or neurons are productively infected by EcoHIV, that this infection is highly efficient (like that of BMM), and we find that two virus clones with different HIV-1 genome backbones [11] and two mouse strains are suitable for use in investigating HIV replication in primary brain cells in culture. This system revealed that EcoHIV infection activates microglia that undergo significant enlargement, indicative of cell activation like that seen during neuroinflammation [28,29,30]. While we do not find productive EcoHIV infection of astrocytes in these cultures, our observation does not rule out the abortive or latent infection of astrocytes, as has been observed in brains of PLWH [1,2].

The mixed brain cell culture employed here contains astrocytes, microglia, and neurons, allowing for the convenient investigation of neuronal defects imposed indirectly by productively infected microglia. Despite fulminant EcoHIV infection observed in microglia in culture, neighboring neurons do not undergo apoptosis, mirroring our findings with highly efficient EcoHIV infection of the brain in mice [14] and the findings of others in HIV-suppressed patients with NCI [34,39]. Recent studies have identified other forms of regulated cell death that may influence HIV neuropathogenesis. Tat protein can induce ferroptosis in primary microglia; ferroptosis has also been implicated in neuropathogenesis in HIV Tg-rats and in PLWH suffering NCI [45]. In addition, elements of pyroptosis were demonstrated in a variety of relevant systems, including in neurons from PLWH, human neuronal cells exposed to HIV Vpr in culture, and in brains of SIV-infected macaques [46]. In future studies, we propose to investigate these important mechanisms in EcoHIV-infected mice. Visualized here in EcoHIV-infected mixed brain culture by MAP-2 staining and automated analysis, the axons increased in thickness, and the dendrites were trimmed in number. The latter is highly reminiscent of the dendritic de-arborization observed in EcoHIV-infected mice with NCI [14,15] and in PLWH who died with HIV-NCI [47], offering a tractable experimental system for investigating the mechanism of this phenomenon. Our future studies will also address changes in dendritic spines and spine subtypes. At both RNA and protein levels, during culture with EcoHIV-infected microglia, neurons reduce expression of SYP, a component of synaptic vesicle endocytosis during signal transmission [48]. Critically, this culture system also allows for the investigation of interventions to HIV infection to assess the effects upon neuropathogenesis, as well as virus replication. ABV, a DNA chain terminator that blocks reverse transcription [38], also blocked EcoHIV infection and prevented dendritic pruning and axonal thickening caused by co-culture with infected cells.

These findings reproduce many observations, employing the culture of various animal or human cell types to investigate HIV replication and neuropathogenesis (reviewed in [49]). Among the studies of the effects of isolated Tat protein upon neuronal viability or dysfunction [50,51] is the report that Tat promotes the degradation of MAP2 in primary rat neurons [52]. MAP2 loss and dendritic pruning are morphological defects clearly associated with cognitive impairment in PLWH [47] and in EcoHIV-infected mice [14]. Mice expressing Vpr in the brain also suffered losses of SYP, suggesting a role of this viral protein in HIV neuropathogenesis [53]. In addition, a recent study demonstrated a correlation between the expression of HIV Nef in brain tissues of PLWH and the extent of neurocognitive disease in these patients [54], suggesting the involvement of Nef in neurocognitive impairment. EcoHIV/NL4-3 and EcoHIV/NDK encode Tat, Nef, and Vpr, along with all other HIV proteins, except gp120 [11]. The brain cell culture system used here may allow for a detailed elucidation of the functions of these proteins in synaptodendritic injury by HIV through targeted mutagenesis of the known functional domains of these viral proteins.

In summary, the results reported here introduce new tissue culture systems amenable to the convenient investigation of HIV pathogenesis and control in the nervous system. Moving from EcoHIV-infected animals to infection in primary tissue culture reduces costs in personnel effort, research reagents, and animal purchase and housing. It also conforms to the NIH policy of replacing living animal subjects with culture models, whenever feasible. We hope that identifying interventions to prevent or ameliorate cellular dysfunction associated with HIV brain disease can be facilitated by the use of these relatively simple and versatile brain cell culture models. 

## Data Availability

Data collected from micrographs are provided in the manuscript.

## Supplementary Materials

The following supporting information can be downloaded at: https://www.mdpi.com/article/10.3390/v16050693/s1. Table S1. Gene sequences of probes and primers for QPCR. Table S2. (for Figure 4) Microglia cell size measurement of EcoHIV/NDK-EGFP-infected cell percentage to compare infectivity of EcoHIV/NDK-EGFP in difference mice strains, C57BL/6 and 129x/SV mice in brain glial cell culture.
