# Supplementary material for: EcoHIV Infection of Primary Murine Brain Cell Cultures to Model HIV Replication and Neuropathogenesis
Source: Viruses. 2024 Apr 27;16(5):693. doi: 10.3390/v16050693 (PMC11125688; doi:10.3390/v16050693)
Supplement: Supplementary file 1 [file viruses-16-00693-s001.zip › viruses-2935325-supplementary.pdf]

Gene Name		Probes (10 μM)	Primers (90 μM)
MLV Env		EcoP 5´- CAGACCAACAGCCACT-3´	EcoF 5´-GGCCAAACCCCGTTCTG-3´<br>EcoR 5´-ACTTAACAGGTTTGGGCTTGGA-3´
Synaptophysin		Roche Universal ProbeLibrary Set, Human #72	F 5´-AACAACAAAGGGCCAATGAT-3´<br>R 5´-TAGCCACATGAAAGCGAACA-3´
Vif	EcoHIV/NL4-3	NLVFQP<br>5´-AGTAGTAATACAAGATAATAGTGA-3´	NLVFQ5 5´-CGCGCACGGCAAGAG-3´<br>NLVFQ3 5´-CTTTGCTTTTCTTCTTGGCACTACT-3´
	EcoHIV/NDK	QVFP<br>5´-AGTAGTAATACAAGACAATAGTG-3´	QVF52 5´-AAGAGGCGAGGGGCAGCGA-3´<br>QVF3 5´-TCTTTACTTTTCTTCTTGGTACTACCTTTATG-3´
Tat	EcoHIV/NL4-3	NLTatP<br>5´- AAAGCCTTAGGCATCTC-3´	NLTatF 5´- CCTAAAACTGCTTGTACCAATTGC-3´<br>NLTatR2 5´-GTCGGGTCCCCTCGGGATTGGGAG-3´
	EcoHIV/NDK	NDTatP<br>5´- AAAGGCTTAGGCATCTC-3´	NDTatF 5´- CCTAGGACTGCTTGTAATAAGTGT-3´<br>NDTatR2 5´-GTCGGGTCCCCTCGGGACTGGGAG-3´
Mouse GAPDH		20x mix (ABI 4352932 E)	

Supplementary Table S1. Gene sequences of probes and primers for QPCR

	129x1/SV		C57BL/6	
Microglia size (μm)	Uninfected	Infected	Uninfected	Infected
Average	35.373	144.571	28.8	163.357
STD error	6.282	23.409	5.304	21.289
t-test				
129x1/SV Uninfected vs.		0.000000213	0.159374	0.000000000103
129x1/SV Infected vs.			0.000000215	0.299825
C57BL/6 Unintected vs.				0.000000000141

Supplementary Table S2. (for Figure 4)
Microglia cell size measurement of EcoHIV/NDK-EGFP-infected cell percentage to compare infectivity of EcoHIV/NDK-EGFP in difference mice strains, C57BL/6 and 129x/SV mice in brain glial cell culture.
